# Supplementary material for: Are carbon emissions trading and green financial instruments synergistic? -Comprehensive quantitative research based on content analysis
Source: PLoS One. 2024 Mar 7;19(3):e0298601. doi: 10.1371/journal.pone.0298601 (PMC10919601; doi:10.1371/journal.pone.0298601)
Supplement: S1 Data — (ZIP) [file pone.0298601.s001.zip › Data/地级市Z_F工作报告.zip!/║■─╧/╘└╤⌠╩╨/└┤╫╘╣1⁄2╓┌║┼í╛┬φ┐╦╩2╛▌═°í┐_2010.docx]

各位代表：

现在，我代表市人民政府，向大会报告政府工作，请予审议，并请政协各位委员和其他列席人员提出意见。

2009年：在攻坚克难、科学跨越中保增长、保民生、保稳定

刚刚过去的一年，是进入新世纪以来发展压力最大、困难最多的一年。面对国际金融危机的空前冲击，全市上下在中共岳阳市委的坚强领导下，深入贯彻科学发展观，大力推进"一化三基"战略，努力践行民本岳阳执政和发展理念，创造性地开展工作，完成了市六届人大二次会议确定的年度目标任务，在连续多年保持经济高位运行的基础上，实现了经济社会又好又快发展。预计全市实现生产总值1211亿元，比上年增长13.5％；财政总收入82.6亿元，增长16.3%；全社会固定资产投资640亿元，增长58.5%；社会消费品零售总额432.2亿元，增长19.5％；城镇居民人均可支配收入15674元，增长12.4%；农民人均纯收入5349元，增长11.2%；城镇登记失业率4.3％；万元GDP能耗下降5.5％，主要污染物排放总量减少2.7％；森林覆盖率36.4％；人口自然增长率5.7‰。主要经济指标增幅高于全省平均水平。

（一）新型工业化取得重大进展。全市规模工业增加值完成576亿元，增长18.9%；完成工业税收67.9亿元，增长66.9%；新增规模企业123家，总数达到1195家。全市工业技改投入230亿元，增长40%，炼化一体化项目加快建设，其中催化重整改造、年产20万吨SBS装置挖潜改造等10个项目建成投产。华能三期五号机组是湖南唯一得到国家发改委核准的火电项目，完成投资5.2亿元。岳纸40万吨超级压光纸、驿通电子、东方雨虹等215个项目竣工投产；催化剂新基地、海螺水泥、桑乐太阳能等158个项目开工建设；小墨山核电、湖南电煤储备基地等项目前期工作有效推进。技术创新迈出新的步伐，建成各类工程技术研究中心21家，组建精细化工和电磁装备制造产学研战略联盟，高新技术产品增加值增长31%，淘汰落后产能企业15家。园区工业发展迅速，其工业增加值占全市规模工业增加值的43.1%。滨湖示范区规划建设全面启动，城陵矶临港产业新区基础设施建设、融资工作全面铺开。岳阳经济开发区被定为国家高新技术创业服务中心，田谷电子信息产业园成为省级产业基地，中国湖南轻工产业园落户湘阴，岳阳印刷工业科技园落户君山。新型工业化主导县域经济发展，县域经济占GDP的比重达到56.3%，非公有制经济比重达到55.2%，分别提高1个百分点和1.9个百分点。继汨罗、华容之后，岳阳县进入全省经济强县，临湘进入全省县域经济发展先进县市区。新型工业化继续保持全省领先地位。

　　（二）新农村建设全面推进。农业生产喜获丰收，粮食总产315.5万吨，增长7.6%；出栏生猪729万头，增长2.8%；水产品产量36.2万吨，保持全省第一。现代农业加快发展，全市农产品加工企业发展到1986家，其中204家龙头企业带动组建农民专业合作组织373个、联基地187万亩。农业生产综合机械化水平达到53.3%。华容和岳阳经济开发区设立小额贷款公司。全市发放小额农户信贷8.6亿元。农村基础设施建设力度加大，投入资金50多亿元，硬化农村公路2030公里，新建农村客运站场12个；完成水利工程5.2万处，清淤整修渠道3100公里，改善灌溉面积19.3万亩；治理水土流失面积40平方公里，实施农村土地整理项目62个；"清洁家园"村民小组增加到2.6万个，新建农村沼气池4.7万口，新解决33.3万农村人口饮水安全问题。新农村建设示范片、示范村分别达到19个和303个。层山安全区、林业血防工程等项目顺利推进。国家级台湾农民创业园落户岳阳县，平江扶贫工作扎实有效。全面推行支农惠农资金"一卡通"发放，财政农林水事务支出15.4亿元，增长17.1%。

　（三）现代服务业加快发展。城陵矶新港开港运营，岳阳港口货物吞吐量突破6000万吨，居全省第一、全国内河主要港口第七。物流业成为新的经济增长点，占GDP的比重达到6.7%。旅游业持续旺盛，全市接待境内外游客1380万人次，旅游总收入增长20％；君山区被评为“中国野生荷花之乡”，平江加义镇、汨罗长乐镇被评为省级特色景观旅游名镇，张谷英村被评为“中国生态文化村”，得胜南路湖鲜美食街被授予“中华湖鲜美食名街”称号；启动盘石洲生态公园、洋沙湖度假中心等旅游项目29个，发展星级休闲农庄19个。积极开展“家电下乡”、“汽车、摩托车下乡”等促销活动，稳定发展房地产和汽车两大市场，汽车销售额增长31.4%，家电销售额增长39.5%，房地产开发建设面积增长43%。引导中介组织健康发展，市直232个社团进一步规范，基本实现“五脱钩”。金融支持经济发展的作用增强，年末各项贷款余额362.7亿元，比年初增加73.9亿元。融资渠道进一步拓宽，年末省内异地贷款余额67亿元，增加9亿元；新增中小企业融资担保公司4家，全市9家担保公司融资担保13.5亿元；汨罗首创退税权质押贷款和再生资源“一会三公司”担保体系，努力破解中小企业融资难题；中科电气、金成实业成功上市，全市9家上市公司融资17.8亿元；保险市场主体发展到32家，开发“三农”保险产品50多个，农业政策性保险理赔8012万元。

（四）城市面貌和基础设施明显改观。全市完成城镇建设项目136个，投入资金29亿元。完成市本级土地利用总体规划和城市总体规划修编、市域城镇体系规划审查。中心城区建成巴陵广场、武广客运专线岳阳站及配套工程；拉通青年中路、建湘路和枫桥湖路，城区路网结构大为改善；全面开展洞庭风光带三期、洞庭大道和五里牌路、花板桥路提质改造；新建续建旧城改造项目14个，新建改造小街巷96条；南湖治污成为全国内湖水环境治理成功范例；新增绿地面积4万平方米；强力推进控违拆违，查处违法建设1255起，拆除9.6万平方米；建成6个污水处理厂。顺利通过国家卫生城市抽查、国家园林城市复查，文明城市创建活动深入开展，岳阳入围新中国成立60年城市发展60强。各县城和中心镇基础设施逐步完善，城镇品位进一步提升。完成交通建设投资61.6亿元，增长2.4倍。武广客运专线岳阳段建成运营；总投资188.7亿元的随岳（随州至岳阳）、岳长（岳阳至长沙）、岳常（岳阳至常德）、通平（通城至平江）、石华（石首至华容）5条高速公路相继开工，完成投资30亿元；G106平江段、S202一期工程、S201岳汨公路、岳荣新二期、进港道路、临鸭公路完成主体工程。完成电力投资12.6亿元，岳阳县、云溪区220千伏和鹿角、公田、古培、城西110千伏变电站等项目投入运行。

　　（五）民生得到持续改善。就业和保障规模扩大，新增城镇就业4.5万人，失业人员再就业3万人，零就业家庭就业援助实现动态清零，农村劳动力转移就业89万人；养老、失业、医疗、工伤、生育保险新增参保20.2万人；城镇居民医疗保险参保达到104万人；新农合参合率达到94.2%，农民受惠3.3亿元。在全省建立首个特困家庭大病医疗慈善救助基金，募集资金1800万元，重点帮助特困群体医疗解困。城乡低保下拨资金同比增加5400万元。五保集中供养人平标准提高400元，达到2400元，分散供养标准提高240元，达到1040元。完成市儿童福利院主体工程，建成岳阳残疾人服务中心。率先在全省实现乡乡有敬老院的基础上，改扩建乡镇敬老院20所，新建50个村级五保之家。帮助洞庭湖区捕捞渔民解困，无房渔民全部安置上岸定居，5594名特困渔民纳入城乡低保。切实解决群众住房困难，新建改建廉租房10069套、45.3万平方米，新增廉租住房租赁补贴6745户；建设经济适用房17.5万平方米。帮助重建、改造和维修农村困难群众住房3280户。岳阳被评为"全省双联和帮扶特困职工先进市"。一年来，我们坚持各项政策和措施进一步向民生倾斜，全年公共财政投入民生85.9亿元，占财政总支出的67.9%。省市实事67项指标全面完成和超额完成。危机之年、困难之年，人民群众得到了更多实惠。

　（六）社会事业协调发展。教育强市迈出坚实步伐，未成年人思想道德教育走在全国前列，建成义务教育合格学校122所，高考二本以上录取率、职业教育和民办教育综合排名全省第一，岳阳县职教中心经验在全国推广；湖南理工学院成功申报硕士点，岳阳职业技术学院入围湖南省示范性高职院校，湖南民族职业学院被国务院授予“全国民族团结进步模范集体”称号。科技创新取得新成绩，顺利通过国家知识产权试点城市验收，3项科技成果获国家科技进步奖。城乡公共医疗服务体系建设加快，改扩建县级医院3座、乡镇卫生院44所，建设村级卫生室71个、社区卫生服务中心14所；甲型H1N1流感等重大传染病防治工作取得成效；岳阳楼区社区卫生“服务零距离、药品零利润、医患零纠纷”模式在全国推广。人口计生工作实现“保先进位创优”目标，君山、云溪进入全省优质服务先进单位。以“汨罗江畔端午习俗”为主要内容的中国端午节，列入世界非物质文化遗产名录，是我国首个入选的节日。《今上岳阳楼》荣获田汉新剧目奖。成功举办“祖国颂”大合唱等一系列庆祝新中国成立60周年纪念活动。全民健身活动丰富多彩。严厉整治安全隐患，安全形势整体平稳好转。组织开展了“扫黑除恶”、“社会治安整治”等专项行动，对各类违法犯罪始终保持严打高压态势，屈原、临湘、君山进入全省综治民调前列，人民群众安全感进一步增强。集中处理重大信访问题，信访工作综合考核排名全省第一。严厉打击传销，严肃查处食品药品违法案件。在全省率先建立医患纠纷第三方调解机制。“扫黄打非”保持全国先进，岳阳被评为“全国见义勇为好城市”、“全省社会治安综合治理工作先进市”。

一年来，全市广泛开展深入学习实践科学发展观活动，加强精神文明建设和民主法制建设，推动经济社会良性发展。承办人大议案、代表建议186件，承办政协建议案、委员提案463件，办结率100%，满意率99%。村民自治巩固发展，社区建设取得新成效。国防教育、国防动员和后备力量建设不断加强。统计、工商、质监、气象、史志、档案、保密、盐业、烟草、老龄、对台、妇女儿童、广播电视、邮政通信、民族宗教、社会科学等各项事业，都有新的亮点、新的进步。

各位代表，我市经济社会发展的显著成绩，是在极为复杂、困难的情况下取得的，确实来之不易，主要得益于以下六个方面：

　　一是全力贯彻上级决策。面对挑战和机遇，全市上下坚决、创造性地贯彻中央保增长一揽子计划和省委、省政府"弯道超车"部署，按照市委"攻坚克难、科学跨越"的要求，出手快、出拳重、措施准、工作实，积极争取国家和省里的项目投资，扩大市场融资，出台相关配套政策，开展“百日行动”，取得应对危机的主动权，推动了各项工作全面落实。

　　二是努力优化发展环境。把解决影响发展环境的突出问题作为保增长的重要举措，在全省率先建立部门行政许可职能集中行使和建设工程项目联合审批、联合验收制度，全面推行投资项目审批代办和限时办结，实施重点企业、重大项目挂牌服务，取消一切未经市委、市政府批准的检查评比，每季度对经济环境进行测评，公布结果。查处"三乱"和影响经济环境的案件26起，取消行政审批事项124项，着力营造高效、务实、透明的发展环境，投资者、经营者对发展环境满意度提高。

三是大力推进项目建设。坚持把扩大投资作为经济增长的第一驱动力，积极开展"项目建设与服务年"活动。各级各部门打破常规服务项目、扩大投资，开工建设城镇以上固定资产投资项目1482个，着力推进88个重点项目建设和65个重大项目前期工作，全年固定资产投资接近前两年的总和，多年为之努力的重大项目逐一得到落实，不仅实现了当前保增长，而且增强了岳阳长远竞争力。

四是合力开展企业帮扶。为抵御国际金融危机对实体经济的冲击，充分发挥政府和市场“两只手”的作用，积极开展联手帮扶企业行动，建立“挂号销号”制度，服务生产经营和项目建设，帮助企业解决融资、用地等实际问题1039个，调处各类影响企业生产经营的矛盾纠纷541起。121家停产半停产企业全面恢复生产，一批优强企业抓住发展机遇，扩大了市场份额。

　　五是强力推进改革开放。坚持解放思想，用改革的办法破解难题，用开放的思维敞开门户促发展。市属国有企业改制完成23家，7800名职工进入社区；企业改制启动完成、融资规模、职工移交是历史上最多的一年。交通体制进一步理顺，11个政府还贷二级公路收费站全部撤除，妥善分流安置干部职工1112名。集体林权制度改革进展顺利，林地发证率达到50.7%。城乡用地增减挂钩试点不断扩大，对缓解用地供需矛盾发挥了积极作用。全面承接产业转移，全年引进内资项目568个，到位资金139.8亿元，增长17.2%；实际利用外资1.3亿美元，增长29.7%；完成进出口总值9.3亿美元，增长9%。22个国家驻华使节参加岳阳第6届国际观鸟节，岳阳被授予"最值得驻华大使馆向世界推荐的中国生态城市"，对外形象和影响力进一步提升。

　　六是着力加强自身建设。适应政府职能转变和市场经济发展需要，加强公务员队伍教育管理，深化忠诚主题教育活动，提高科学谋事干事水平。严格政府绩效考核，对行政效能定期组织公开测评，认真执行"有错无为问责"，不换状态就换人，辞退2名公务员，行政处分225人，进一步严格政风、严明政纪、严肃政令。推进政务公开，加强廉政建设，在土地出让、工程招标、产权交易、政府采购、资金拨付、人事安排等方面创新制度、公开办事、接受监督，完成财政、投资和领导干部经济责任审计项目617个，反腐倡廉保持全省先进，努力提高政府公信力。

各位代表，回顾一年来的工作，我们深感每一项工作的推进，每一项成绩的取得，都离不开市委的坚强领导，市人大、市政协的监督支持，凝聚着全市人民的智慧和力量。在此，我代表市人民政府，向全市人民，向所有关心、支持和参与岳阳改革建设的人们，表示崇高的敬意和衷心的感谢！

我们也清醒地看到，全市经济社会发展面临的困难和问题还不少。主要是：三产业比重偏低，财政总收入占GDP的比重偏低，高新技术产业增加值占工业增加值的比重偏低，经济结构有待进一步优化；农业基础相对薄弱，农民持续增收乏力；中小企业融资难问题还没有得到有效解决；就业、环保、安全生产等压力加大，维护稳定的任务还很艰巨；一些部门职能交叉、权责脱节，执法不公、政令不畅、效率不高等问题和消极腐败现象还不同程度存在，政府工作与形势的要求和人民的期望还有差距。对此，我们一定高度重视，采取切实措施加以解决。

　　　2010年：提速、升级、增效、惠民全面完成和超额完成“十一五”规划任务

今年是实施"十一五"规划的最后一年，也是应对国际金融危机的关键之年，面临的形势仍然极其复杂，外部需求萎缩，内生动力不足，转变发展方式任重道远。但总体来看，今年经济发展形势会好于去年，世界经济有望恢复性增长；国家继续实施积极的财政政策和适度宽松的货币政策，更加重视经济结构战略性调整、中小城镇发展和民生改善，为我市加快发展带来新的契机；特别是近年来我市一批重大项目强力推进，经济基础得到有力夯实，发展态势良好。我们既要看到面临的复杂形势，更要坚定信心、抢抓机遇、乘势而上，推进科学发展。

今年政府工作总体思路是：坚持以科学发展观总揽全局，全面贯彻党的十七大、十七届三中四中全会和中央、省委经济工作会议精神，大力实施“一化三基”战略，深入践行民本岳阳执政和发展理念。按照“提速、升级、增效、惠民”的要求，着力加快经济发展，做大经济总量；着力转变发展方式，优化经济结构；着力提高经济增长质量和效益，增强发展竞争力；着力改善民生，促进社会和谐，加快实现“产业兴旺、城乡秀美、文化繁荣、民生殷实、社会和谐、走势强劲”的富民强市目标。

今年主要预期目标是：全市生产总值增长11%；财政总收入增长13％;规模工业增加值增长15％；全社会固定资产投资增长30％；社会消费品零售总额增长16％；城镇居民人均可支配收入和农民人均纯收入分别增长10％和9％；城镇登记失业率控制在4.5%以内；人口自然增长率控制在6‰以内；万元GDP能耗下降4.2%，SO2、COD排放总量分别削减1.18%、0.67%，全面完成和超额完成"十一五"规划任务。

今年的政府工作，重点从八个方面推进：

一、大力推进扩大内需

继续保持投资、消费快速增长势头，拉动经济增长，增强发展动力和后劲。

加快基础设施建设。道路交通：开工建设荆岳铁路。全面推进随岳、岳常、岳长、通平、石华高速公路建设，启动杭瑞高速临湘至岳阳段项目。力争S308湘阴段和平江段、羊楼司连接线、铁桃公路、汨罗江大桥及接线工程等项目竣工，加快S202二期、S306华容段、S306岳阳至平江段项目建设，启动炼化公路、荣鹿公路等干线公路和鲇鱼须大桥项目。港口码头：力争洞庭湖水上应急救援指挥中心一期工程竣工运营，加快二、三期工程建设。抓好长航综合码头、恒阳化工储运码头建设，开工建设临湘鸭栏码头，统筹抓好塔市驿、鹿角和汨罗港、漕溪港码头二期项目，加快长江航道疏浚。水利：积极应对洞庭湖低水位危机影响，组建洞庭湖气候中心。扎实推进洞庭湖综合治理，加快蓄洪安全工程建设，完成钱粮湖层山安全区围堤工程、钱粮湖大垸堤防加固华容县项目，完成12处大型排涝泵站更新改造，扎实做好钱粮湖、大通湖东、城西、屈原、建新、麻塘等堤垸防洪工程建设和华容河整治项目前期工作，提升防洪保安能力。电力：扩建2个220千伏变电站，新建和扩建4个110千伏变电站，新建改造4个城配网项目，加快中心城区电缆入地改造。燃气：建成岳阳至云溪长输管线，中心城区新建2座天然气供应站，推进县级燃气管线、气站建设，改善居民用气条件。机场：抓紧做好岳阳支线机场前期工作，力争进入国家、省“十二五”规划。

　　加快城镇扩容提质。推进新型城镇化，进一步拉动投资、激活消费，发展城市经济，改善人居环境。推动城市规模扩张。中心城区坚持“东扩、南延、北靠、西连、中提”发展战略，以“三跨一连”拉开大城市格局：开发金凤桥新片区，建设皇姑塘立交桥、市体育中心和登旺汽车站，续建金凤桥路，跨107国道发展；加快建设洞庭湖国际公馆，拓宽加固湖滨防洪大堤，续建园艺路，跨南津港大堤发展；凭借荆岳铁路和岳常高速，提质改造洞庭大道君山段，跨湖发展；续建联港路、望岳路和冷水铺路，连接临港产业新区。引导中心城区工业“退二进三”，拓展城市发展空间。完善城市功能配套。完成洞庭风光带三期、环城水系和中心城区近期建设专项规划。加强城市路网建设，拉通德胜北路，启动青年东路、花果畈路和环城公路建设，改造巴陵中路非机动车道，续建枫桥湖路楼前广场，全面提质改造中心城区小街小巷。加快环南湖截污管网建设和王家河综合治理。加快城市公交站场建设，新建2个大型渣土专业处置场和一批公厕、垃圾站，增强城市服务功能。塑造城市特色风貌。提升城市建筑、绿化、雕塑等规划建设水平；加快城区主干道两侧建筑立面改造；加强铁路沿线周边环境整治；抓好门店招牌、街道门牌、户外广告管理，提高城市美观度，充分展示岳阳湖光山色美、古今交融美、现代动感美。创新城市管理机制。坚持政府主导，推进城市管理市场化、数字化、精细化和社区化，建立两级政府、三级管理、四级网络的城管服务体系。加快城市管理重心下移，构建社区"三室三中心"管理服务和社区志愿服务网络体系，推动"12319"城市管理热线与社区管理有机结合，把社区建成管理有序、服务完善、文明和谐的生活共同体。深入创建国家交通管理模范城市。推进城乡一体化进程。加快县乡总体规划修编，逐步实现县城、建制镇控制性详细规划全覆盖。坚持"一核三圈"城镇梯级发展，以市区为核心，加快中心城区周边小城镇、县城和市域周边小城镇"三圈"建设，重视发展湘鄂赣边界城镇，引领区域经济发展。

　　加快旅游业发展。以文化旅游、生态旅游、休闲旅游为主题，打响岳阳旅游品牌，形成新的消费增长极。建设大景区。推动楼岛湖核心景区整合提质，力争成功申报东洞庭湖国家级旅游度假区、慈氏塔国家级文物保护单位。抓好屈子文化园、龙窖山古遗址景区、洞庭山庄、市游客服务中心等重大旅游项目建设。实现5A景区、五星酒店"零"的突破。打造大产品。发展团湖野生荷花、西塘桃花生态观光游，张谷英、左宗棠民俗体验游，龙舟竞渡、公田温泉康体游，平江漂流探险游，农家乐休闲度假游，开发参与型、娱乐型、文化主题型旅游产品和工业旅游走廊。依托特色旅游产品，策划精品线路，扩大旅游消费。形成大合作。以武广客运专线运营为契机，主动对接港澳台、珠三角和武汉等旅游热点市场、旅游大公司，促进旅游一线通和旅游加速。开展大促销。加大媒体、网络、节会、文化营销力度，在更大范围内参与竞争，提高岳阳旅游知名度。

加快新兴服务业发展。把服务业作为扩大消费、调整结构的重要着力点，进一步提升传统服务业，做强新兴服务业。大力发展现代物流。建设烟草进出口基地及配送中心、粮食物流交易中心、君山农副产品配载物流中心，支持物流园区加快发展。建设生猪机械化定点屠宰场，改造一批农贸市场，推进"万村千乡市场工程"，农家店覆盖90%以上的行政村。依托现代信息技术，大力发展物联网，加快完善社会化、专业化物流服务体系。积极发展现代金融。开展"金融服务年"活动，创新金融产品，加快信用担保体系建设，努力保证重点建设项目和中小企业贷款需求。支持市商业银行做大规模，新建2家支行。力争新引进1家股份制商业银行，新组建1家小额贷款公司。支持胜景山河、凯美特上市，推动岳阳恒立重组复牌。充分利用好上市公司资源，扩大再融资能力。加快发展文化创意产业。推动文化资源产品化、文化产品产业化和文化产业市场化，大力发展影视传媒业、演艺娱乐业、图书音像业、广告设计业、文化会展业。鼓励和支持非公有制企业进入文化产业，繁荣文化市场。引导中介服务业规范发展。重点发展市场交易组织和公证、代理、信息技术服务机构，规范市场交易行为，降低市场交易成本。促进房地产业健康发展。大力发展楼宇经济，加大房地产开发力度，加强物业管理，加快旧城改造步伐。实施控违拆违属地管理和乡村规划许可机制，严厉查处违规建房，严禁行政企事业单位集资建房和城市规划区内村民零星分散建房，规范房地产开发秩序。

二、大力推进新型工业化

以新型工业化为主导，开展"产业发展升级年"活动，提速科学发展，推动产业转型升级，加快构建具有岳阳特色、富有竞争力的现代产业体系。

做大产业集群。大力组织实施"千百十"工程，重点培育2个千亿元产业、15个百亿元基地、100家主营业务收入过十亿元或"专精特新"的"小巨人"企业，年内新增规模企业100家以上。做强石化、食品产业。加快发展特色化工产品、精细化工产品和高分子功能材料。支持长炼原油扩能升级改造、巴陵石化"五改七建一配套"、催化剂新基地、东方雨虹二期等项目建设。推进云溪精细化工园和儒溪农药化工园加快发展。支持正虹发起组建国家生猪产业技术创新战略联盟，依托岳阳福润等企业，引领肉类加工业扩张发展；依托巴陵油脂、鲁良新元和长康实业等企业，推动油脂产业加快发展；依托胜景山河、华润雪花、岳阳楼酒业等企业，促进岳阳黄酒、啤酒、白酒、保健酒形成规模和品牌；依托农产品资源优势，壮大茶叶、大米、蔬菜、水产加工业。做大机械制造、能源产业。抓好化工、造纸、港务、船舶、工程和农机装备制造业项目的招商引进。支持中科电气、中天科技、天一科技等上市企业扩张发展。谋划创建农机产业园。确保华能三期两台机组建成投产，力争小墨山核电、湖南电煤储备基地和平江抽水蓄能发电等重大前期项目列入国家、省"十二五"规划。做优生物医药、造纸印刷产业。抓好生物医药工业园发展，支持科伦双室软袋生产线等项目建设，逐步形成以中药材资源为依托、以高新技术为支撑的生物医药产业集群。加快岳阳印刷工业科技园建设，依托泰格林纸、丰利纸业等拉长产业链，向纸制品深加工和印刷、包装业延伸。提升纺织、建材产业。支持规模纺织企业发展织布、印染、服装业，支持水泥、陶瓷等建材业创新工艺，促进产品升级换代。培育新兴产业、配套基地。以循环经济、绿色经济、低碳经济为导向，加快发展环保、信息等新兴产业。深化汨罗工业园和泰格林纸循环经济试点。支持岳阳经济开发区和汨罗、湘阴、华容电子信息园加速扩张。确保桑乐太阳能一期工程竣工投产，年生产能力达到40万台。紧盯发达地区产业转移与国内外优强企业、湖南千亿产业和岳阳优势产业，发挥比较优势，提高产业配套能力。支持湘阴等地对接长沙轻工、机械制造和电子信息产业，建设配套基地。

推动科技创新。以技术进步促进发展方式转变，完成技改投入260亿元。加快科技创新平台建设，建成国家磁力设备质量监督检验中心，创建5家工程技术研究中心或重点实验室，组建生物医药、建筑陶瓷产学研战略联盟。组织实施一批共性关键技术和重要装备开发项目，为企业发展提供技术支持。推进质量、品牌和标准化建设，力争全市具有自主知识产权的品种达到360种，新认定高新技术企业20家。加大人才培训、引进、选拔、使用力度，培养一批职业经理人和科技领军人才，完善人才激励机制，形成地域经济发展的核心推动力。

大力建设临港产业新区。强力推进临港产业新区建设，使之成为岳阳乃至湖南加强与长三角联系、深化与东部沿海合作、促进与中原腹地经贸交流的重要基地。将港区规划建设区、规划控制区分别调整到69平方公里、100平方公里。加大财政、金融、用地支持力度，集中抓好基础设施建设和项目引进，促进新港与老港融合、临港产业新区与城陵矶片区对接。授予临港产业新区市级综合经济管理权限，畅通项目建设绿色通道，确保今年临港产业新区建设取得明显成效，力争5年以后工业增加值过100亿元，建成航运物流园区、新兴产业园区和循环经济园区，推进沿江大工业区建设。编制滨湖示范区655平方公里规划，加快建设芙蓉大道湘阴段，抓好汨罗再生资源二期市场建设，做好推山咀码头前期工作，主动接受长株潭辐射，推动滨湖示范区竞相发展。

加快工业园区发展。加强县域经济产业布局和规划指导，支持各地依托产业基础、资源禀赋和区位优势，大力发展特色产业和民营经济。努力促进园区基础设施配套化、发展方式"两型"化、产业关联化、企业集群化、管理科学化、风貌城市化，增强市场竞争力和可持续发展能力。支持岳阳经济开发区加快发展，使其真正成为产业高地和经济增长极。支持上市公司和有实力的企业创办工业园区。

三、大力推进新农村建设

突出农村基础设施建设和现代农业发展，促进农业增效、农民增收、农村繁荣。

加快农村基础设施建设。完成通乡公路100公里、通畅工程1000公里，改扩建临湘客运中心站和华容汽车站，启动汨罗新汽车站和10个农村客运站场建设，加强农村公路管养，防范超载行为。抓紧实施环洞庭湖基本农田建设工程，推进农村土地综合整治。完成70座病险水库除险加固，加强灌区渠系改造、山塘清淤等农田水利建设。加快平江、岳阳县等县（市）农网改造，建成7个农村主电网和11个配电网。加快农村偏远地区通讯、邮政等设施建设，全面完成"十一五"广播电视村村通工程。

提高农业产业化水平。着力构建农工贸一体化经营体系。加快基地建设。提高大宗农产品生产能力，重点抓好10个水稻万亩优质高产示范片，君山和华容蔬菜千亩示范片，君山银针茶、临湘黑茶与湘阴、平江有机茶标准化生产示范基地建设，加快临湘楠竹和平江油茶产业开发。新建5个水产健康养殖小区。力争5亿斤粮食产能基地成功立项，加快大型商品粮基地建设。新增无公害农产品、绿色食品和有机食品认证种类40个，新增农产品地理标志登记2个，提升规模型、标准型农业水平。扩大精深加工。以工业理念谋划农业发展，提高农产品的品种优质率、加工转化率和市场占有率。加快建设台湾农民创业园，推进湘阴和君山全国农产品加工示范基地、平江和华容全国农产品加工创业基地建设，拓展产业化经营，力争农产品加工增加值增长20%。健全服务体系。加强动植物疫病防控、农产品质量监管，健全农产品市场信息化和农机社会化服务体系。力争4个县进入全国农技推广示范县。大力发展各类专业合作组织，实施"绿色证书"培训工程，培养一批农村经纪人、专业合作组织经办人和农村科技示范带头人。

促进农民持续增收。认真落实各项惠农政策，加强惠农资金发放监管，建立稳定的财政支农投入增长机制，努力提高农村居民特别是低收入群体收入水平。大力发展劳务经济，增加农民务工收入。增强农村金融服务能力，大力开展农业开发和农村基础设施建设中长期政策性信贷业务，加快培育村镇银行、贷款公司和农村互助社。扎实抓好农业保险，减少受灾损失。深入开展"万企联村、共同发展"活动。抓好58个扶贫点开发式扶贫，推进平江、岳阳县"两项制度"衔接试点，争取6万农村低收入人口得到国家扶贫开发项目的支持。严格落实国家粮食安全政策，搞好粮食收购、储备和市场管理。

四、大力推进生态建设

牢固树立生态环境就是竞争力的理念，深入开展全国绿化模范城市和全国环保模范城市创建活动，促进人与自然和谐相处。

　　广泛开展造林绿化。增加森林碳汇，提高全市森林覆盖率和城市建成区绿化覆盖率。积极开展"森林进城"、"身边增绿"、"三边造林"和全民义务植树行动，完成造林20万亩。建设森林城市，重点抓好沿洞庭湖、环南湖和京珠高速岳阳连接线三条风光带建设，抓好金鹗山、珍珠山、南湖和龙山四个森林公园建设，抓好洞庭大道、南湖大道、岳阳大道、金鹗路、巴陵路五条林荫大道建设。保护森林资源和东洞庭湖湿地资源，加强森林养护，严格林业和园林执法，巩固绿化成果。

　　继续推进流域治理。推进洞庭湖、湘江、汨罗江、南湖综合治理，筹划东风湖、芭蕉湖综合整治项目，抓好洞庭湖、新墙河等重点水域水利血防整治工程，力争启动游港河、云溪河等11条中小河流综合治理。抓好平江、岳阳县农业综合开发水土保持项目。建成岳阳经济开发区、云溪区、岳阳县和平江工业园4座污水处理厂，提高垃圾无害化处理率。治理农村面源污染和生猪养殖大户污染，推进"清洁家园"行动，推广使用沼气和太阳能，有效处理农村白色污染和废弃农药瓶。加强铁山库区生态保护。完成农村20万人饮水安全工程建设。

　　严格实施节能减排。大力实施清洁生产，促进"环保惠民"。认真落实"环保第一审批权"、"三同时"和排污许可证制度，将有限的排放总量指标用于发展优势产业。加快节能减排重点工程建设，强化节能减排责任，严格限制新上高耗能、高污染企业。强化陶瓷、造纸、化工、采矿等企业污染治理，淘汰一批落后产能。积极推广建筑、交通、商业、民用等领域节能。强化环境执法监管，确保排污企业达标排放，力争中心城区空气质量优良率达87%以上。让岳阳的天更蓝、水更清、山更绿，努力建设秀美岳阳、生态岳阳、宜居岳阳。

五、大力推进改革开放

以更加宽广的眼光、更加开放的姿态、更加灵活的政策，加快改革开放进程。

促进改革实现新的突破。重点抓好七项改革：投融资体制改革，根据"政府主导、市场运作、财政扶持、自求平衡、推动发展"的原则，抓好市城建投、教建投、港建投、交建投、国资公司和旅游发展公司等投融资平台建设，严格按照现代企业制度规范运作，履行好政府公共产品投资者职能，加快公共事业发展，防范财政风险。国有企业改制，启动21家市属国有企业改制，基本完成改制任务。林权制度改革，全面完成集体林权制度主体改革任务，推进配套改革，建立林业要素市场，创新林业管理发展机制。城乡统筹改革，突出抓好平江农村社会养老保险试点；探索失地农民社会保障机制，重点解决城郊失地农民社会养老保险问题；放宽城镇户籍限制，吸纳有条件的农民工特别是新生代农民工转为城镇居民。医药卫生体制改革，深入推进基本公共卫生服务均等化，实施城乡居民和农民工医疗保险试点、岳阳楼区基层医疗卫生机构综合配套改革试点，启动3个县区基本药物制度改革试点。抓好公立医院、企业医院的制度与管理创新，加强民营医院监管。文化体制改革，加快推进国有文化单位和公益性文化事业单位改革，创新文化管理机制。政府机构改革，按照省政府批准的改革方案，推进市县两级改革到位。

推动开放迈上新的台阶。构建大通关格局。建立岳阳与长沙、上海、宁波、武汉"四关联动"快速通关模式。力争临港产业新区成功申报国家级出口加工区，实现保税功能叠加。扩大招商引资。主动对接中央省属企业、知名企业和关联企业，瞄准国内外500强企业和投资公司、私募基金等，上门招商，以商招商。鼓励金融机构、工商联、海联会、行业商会和中介组织参与招商。积极参加"上海世博会"、"台湾湖南周"等重点招商引资活动。力争全年引进内资140亿元、外资1.3亿美元以上。加强开放交流合作。加强与长三角、珠三角等地联系，积极承接产业转移。支持有实力的企业开展国际化经营，扩大境外工程承包和劳务输出，发展外包服务。吸引外国使节、知名人士、海内外客商来岳考察交流、投资创业。

六、大力推进民生改善

加快公益事业发展和民生实事办理，让人民群众共享发展成果。

扩大就业和社会保障。把就业作为改善民生的第一要务，强化就业公共服务，进一步完善就业援助制度，整合部门就业培训资源，重点帮扶就业困难群体。继续实施"全民创业"行动，以创业带就业。新增城镇就业3.9万人、失业人员再就业2.7万人，零就业家庭动态就业援助达到100%，新增转移农村富余劳动力7万人以上。抓好"五大保险"征缴扩面，新增参保17.7万人。提高城乡低保救助标准和医疗保障水平，完善大病医疗救助体系。新建2所县级中心敬老院，创建10所示范敬老院。启动市精神病院等项目建设。积极发展社会福利、慈善捐赠和老龄、残疾人事业。

大力发展教育事业。坚持"教育强市"战略，强化义务教育保障，优化城乡教育布局，促进教育资源均等化。新建市第十五中学，改造160所校安工程学校和35所农村初中，建设86所义务教育合格学校。进一步加强师德和青少年思想道德教育。重视规范幼儿教育，鼓励民间助学办学。发展高等教育，增强高校综合办学实力，积极申报湖南民族师范专科学校。发挥高职院校龙头作用，整合职业教育资源，加大产业工人与农民工技能培训力度，组建石化等4个专业技术人才培养基地，服务地方经济。以岳阳广播电视大学为平台，整合有关资源，大力发展社区教育，在岳阳楼区启动社区教育试点。

扎实抓好医疗卫生和人口计生工作。加快市公共卫生应急救援指挥中心、市妇女儿童医院建设，改造新建5所县级综合医院、6所乡镇中心卫生院、12所社区卫生服务中心。抓好重大传染病防控，加强食品安全综合监管，提升公众饮食与用药安全水平。做好全国第六次人口普查工作。稳定低生育水平，整治违法生育和"两非"行为。全面推行计划生育手术并发症患者扶助治疗试点，抓好全国流动人口"一盘棋"服务管理。

构建公共文化体育服务体系。坚持"文化惠民"，力争50%以上的乡镇建立文化站，新建"农家书屋"556家，积极发展群众文化活动。建设市广电服务中心和市档案馆。深入开展全国文明城市创建活动，积极创建文明社区、文明村组和文明单位。力争在省第十一届运动会上取得好成绩，积极申办省第十二届运动会，抓好180个行政村的农民健身工程。

加快保障性住房建设。创新廉租房、经济适用房建设和分配模式，提高货币补贴的比例，推进城市直管公房和棚户区改造。新增廉租住房8万平方米、经济适用住房15万平方米。改造农村危房1000户。完成831户洞庭湖区捕捞渔民危房改造。探索共有产权房建设模式，帮助更多的人改善住房条件。把改善民生作为政府一切工作的出发点和落脚点，确保新的一年为人民群众办更多实事、让人民群众得更多实惠。

七、大力推进科学管理

　　力争重点领域和关键环节实现管理的精细化，促进政府工作规范有序、公开透明、务实高效。

　　优化决策管理。加强决策调研、咨询和听证，规范决策程序；注重政务信息收集、分析与整理，完善决策办法；把公开透明原则贯穿决策的全过程，加强决策监督。突出决策选优，努力使政府决策更加科学、更顺民意。认真总结“十一五”规划的执行情况，广泛凝聚全社会的智慧，科学编制“十二五”规划。

　　强化政府投资项目管理。简化项目报建手续，优化投资评估、资金评审程序。积极推进项目代建制，推行重大项目设计施工总承包招标。加强安全和质量管理，坚决杜绝"豆腐渣"工程。对重大项目从立项到竣工全过程跟踪审计，优化工程设计和组织，降低投资成本，严防违法违规操作。

完善财税管理。认真抓好财政"省直管县"改革和省以下财政体制调整，进一步深化部门预算改革，扩大国库集中支付范围，加强政府采购监管，开展财政绩效评价。培植壮大财源，促进财政工作迈上新台阶。加强收入管理，进一步推进综合治税，重点打击涉税违法行为。抢抓政策机遇，争取更多财力支持。加强预算管理，厉行节约，严控临时性追加，把更多的资金投向公共服务和民生领域。

　　规范国有资产管理。加强土地、矿产资源管理。全面完成县、乡两级土地利用总体规划修编；严格保护耕地；探索建立建设用地增减挂钩指标、耕地补充指标市域内公开交易制度；依法依规清收闲置用地；全面推行土地、矿权网上招拍挂；整顿规范砂石等矿产资源开采。加强国有及国有控股、参股企业管理，建立健全国有企业治理机制和国有资产管理、评估、转让、监督体系，强化国有企业业绩考核，实现国有资产保值增值。

　　加强社会与安全管理。致力于维护社会公平正义，致力于维护社会公共秩序，创建国家社会治安模范城市。深入开展"打黑除恶"、打击"两抢一盗"、禁毒扫黄等专项斗争。强化安全生产监管，整治安全隐患，坚决遏止重特大事故发生。加强矛盾纠纷排查化解，做好信访和人民调解工作，有效化解不安定因素。大力推进社会诚信体系建设，依法打击传销、制假售假、商业欺诈和逃废债务行为。推进应对自然灾害、事故灾难、公共卫生事件和社会安全事件应急体系建设，提高防灾减灾和预防处置突发事件的能力。积极推进国防动员和后备力量建设，深入开展双拥共建，加强保密和国家安全教育。

八、大力推进政府自身建设

　　面对新形势新任务，进一步加强政府自身建设，始终保持奋发有为状态，努力建设人民满意政府。

　　坚持依法行政。认真落实各项法律法规，深入开展法制宣传教育。进一步规范自由裁量权，推进行政综合执法，加强执法经费保障，规范执法。严厉打击扰乱社会公共秩序、损害公共利益的行为。全面执行人大及其常委会的决议，依法向人大及其常委会报告工作，自觉接受监督。积极支持人民政协履行政治协商、民主监督、参政议政职能，主动听取民主党派、工商联和无党派人士的意见和建议。提高议案、建议和提案办理质量。加强社区等基层组织建设，促进村民自治。执行好民族、宗教、侨务和对台政策，支持工会、共青团、妇联等群团组织依照法律和各自章程开展工作。

　　切实优化发展环境。努力营造开放包容的人文环境、规范高效的政务环境、公平公正的执法环境，切实做到为市场主体和人民群众服务。深化政务公开，加强项目服务，全面推行一票制收费。优化办事流程，努力做到群众办事一看就明白、一听就清楚、一次就办完。加快推进电子政务建设，推行网上办公和网上服务，提高办事效率。重视行政效能投诉，启动电子监察，对扰乱发展环境的人和事，坚决查处，严厉打击，决不姑息。　　加强公务员队伍建设。大兴学习调研之风，深入开展"两个忠诚"主题教育活动，深入基层问政于民、问需于民、问计于民。大兴务实高效之风，把工作重点放在多为基层群众办实事、多为工程项目搞协调、多为企业解难题上，聚精会神搞建设，一心一意谋发展。大兴廉洁从政之风，加强执法监察、廉政监察和审计监督，坚决查处腐败分子。推进政府绩效考核，完善公务员激励约束机制，努力建设一支政治上靠得住、工作上干得好、作风上过得硬、人民群众信得过的高素质公务员队伍。

　　各位代表，新的一年，政府工作任务繁重，我们深感责任重大。让我们高举中国特色社会主义伟大旗帜，深入贯彻科学发展观，按照省委、省政府部署，大力推进“一化三基”战略，在中共岳阳市委的坚强领导下，进一步解放思想，开拓创新，团结进取，奋勇争先，为实现民本岳阳科学跨越而努力奋斗！
